# From cadaveric and animal studies to the clinical reality of robotic mastectomy: a feasibility report of training program

**DOI:** 10.1038/s41598-021-00278-7

**Published:** 2021-10-26

**Authors:** Jeea Lee, Hyung Seok Park, Dong Won Lee, Seung Yong Song, Jonghan Yu, Jai Min Ryu, Soong June Bae, Dea Hyun Lew, Seung Il Kim, Antonio Toesca

**Affiliations:** 1 Department of Surgery, Yonsei University College of Medicine, 50-1, Yonsei-ro, Seodaemun-gu, Seoul, 03722 Republic of Korea; 2 Department of Plastic and Reconstruction Surgery, Yonsei University College of Medicine, Seoul, Republic of Korea; 3 Division of Breast Surgery, Department of Surgery, Samsung Medical Center, Sungkyunkwan University School of Medicine, Seoul, Republic of Korea; 4 Department of Surgery, Gangnam Severance Hospital, Yonsei University College of Medicine, Seoul, Republic of Korea; 5 Division of Breast Surgery, European Institute of Oncology IRCCS, Milan, Italy; 6 Present Address: Department of Surgery, Uijeongbu Eulji Medical Center, Eulji University, Gyeonggi-do, Korea

**Keywords:** Endocrinology, Medical research

## Abstract

Robotic nipple-sparing mastectomy (RNSM) provides better cosmetic outcomes and improves the quality of life of women with breast cancer. However, this has not been widely adapted due to the lack of well-structured training programs. The present study aimed to report the establishment of cadaveric and animal skill laboratory training programs for RNSM and the participants’ perception on the training programs. We performed 24 RNSMs using 11 cadavers and one porcine model. Then, the skill laboratory characteristics were reviewed. Five trainers and 10 trainees participated in the programs. The first four cadaveric RNSMs with latissimus dorsi flaps and implants were performed using the da Vinci Si® system. We performed 14 and six RNSMs using the Xi® and SP® systems, respectively. The scores for questionnaires on the satisfaction with the training consisted of the trainees’ perceived goals in attending the course, teaching/learning environment, and teaching staff performance. The scores were excellent. Cadaveric or porcine RNSM skill laboratory training may be essential programs that can provide safe and efficient training.

## Introduction

Surgery requires practice. Therefore, senior surgeons provide education on the state-of-the-art operative skills for surgical trainees, including junior surgeons, fellows, and residents. The traditional and most effective way to learn surgical skills is through performing the operation itself with appropriate supervision from experienced senior surgeons. However, in modern healthcare systems, the number of operations performed by trainees may be limited by a lack of resources, including facilities, supervisors, and education time. Furthermore, case selection difficulties, non-cooperative patients, and post-operative complication risks are major surgical education concerns in clinical practice. Therefore, well-designed animal or cadaveric skill laboratories have been used in educational programs for surgical trainees^1,2^.

Recently, several surgeons have developed a robotic nipple-sparing mastectomy (RNSM) technique^3–7^, which provides better cosmetic outcomes and improves the quality of life in women with breast cancer or *BRCA* mutations^8–10^. Moreover, the use of the robotic surgical systems’ 3-dimensional high-resolution cameras, flexible robotic instruments, and ergonomic devices can decrease the surgeons’ workload^11^. However, with the exception of the few aforementioned surgeons, it is difficult for breast surgeons to gain experience using the robotic surgical systems alone. Nevertheless, the use of robotic surgical systems in breast surgery remains an emerging area of surgical techniques. Most surgical training centers lack proper educational programs for breast surgery fellows.

Various surgical fields have incorporated surgical training with cadaveric or animal skill laboratories for endoscopic or robotic surgery^1,2^. These skill laboratories provide a simulation of the real clinical operations^12^. Studies have suggested that these skill laboratories supplement the surgeons’ learning experience^13^. However, there have been few attempts at cadaveric or animal skill laboratories for RNSM. Therefore, this study aimed to report the establishment of cadaveric and animal skill laboratory training programs for RNSM and the participants’ perception on the training programs.

## Methods

We performed 24 RNSMs in 11 cadaveric models and in one live porcine model from December 2013 to November 2020. All of the models were female. The porcine model was 14 weeks old and weighed 40 kg. From 2013, skill laboratory training using the da Vinci Si® and Xi® surgical systems (Intuitive Surgical, Sunnyvale, CA, United States) was performed at the Severance Minimally Invasive Surgery Center, Seoul, Korea. On the other hand, skill laboratory training using the da Vinci SP® surgical system (Intuitive Surgical, Sunnyvale, CA, United States) was performed in November 2018 in Medizin im Grünen, Berlin, Germany.

A total of 15 surgeons, thirteen of whom were breast surgeons and two were plastic surgeons, participated in the skill laboratory training. The first three breast surgeons and two plastic surgeons participated in the initial development phase of multi- or single-port systems. There was no education process by a supervisor since this was the first attempt at the cadaveric RNSM program. Therefore, the five surgeons did not answer the questionnaire. After the initial development phase, one surgeon who participated in the initial development phase supervised the trainees during the training phase. All surgeons participated in the dry skill laboratory training, in which a simulator for the basic robotic surgical system principles and techniques was used. For the initial multiport system development phase, a multidisciplinary team discussed various approaches to RNSM with immediate reconstruction. Hence, a report on breast reconstruction using multiport systems was reviewed^14^. However, due to the lack of reports on the initial multiport system development phase in RNSM, other procedures, including endoscopic mastectomy and robotic thyroidectomy, were adapted. Therefore, the cadaveric skill laboratory training used non-insufflated RNSM with a robotic thyroidectomy self-retractor for the initial multiport system development phase^5^. Detailed methods of the initial phase for a single port system were described in a previous study^15^. For the training phase, one or two lectures, which included an introduction to porcine anatomy and minimally invasive breast surgery and standard RNSM operating procedures, were delivered just before the procedures were performed. Each cadaver or porcine model was shared by two to three trainees. For the robotic procedures on mastectomy, there was no specific inclusion or exclusion criteria for the animal model except for one inclusion criterion: being a female porcine. Seven of 10 participants responded to the questionnaire promptly after the training program, and two participants answered the questionnaire within a month. Only one participant answered the questionnaire within 3–4 months after completing the training.

**Figure 1:**
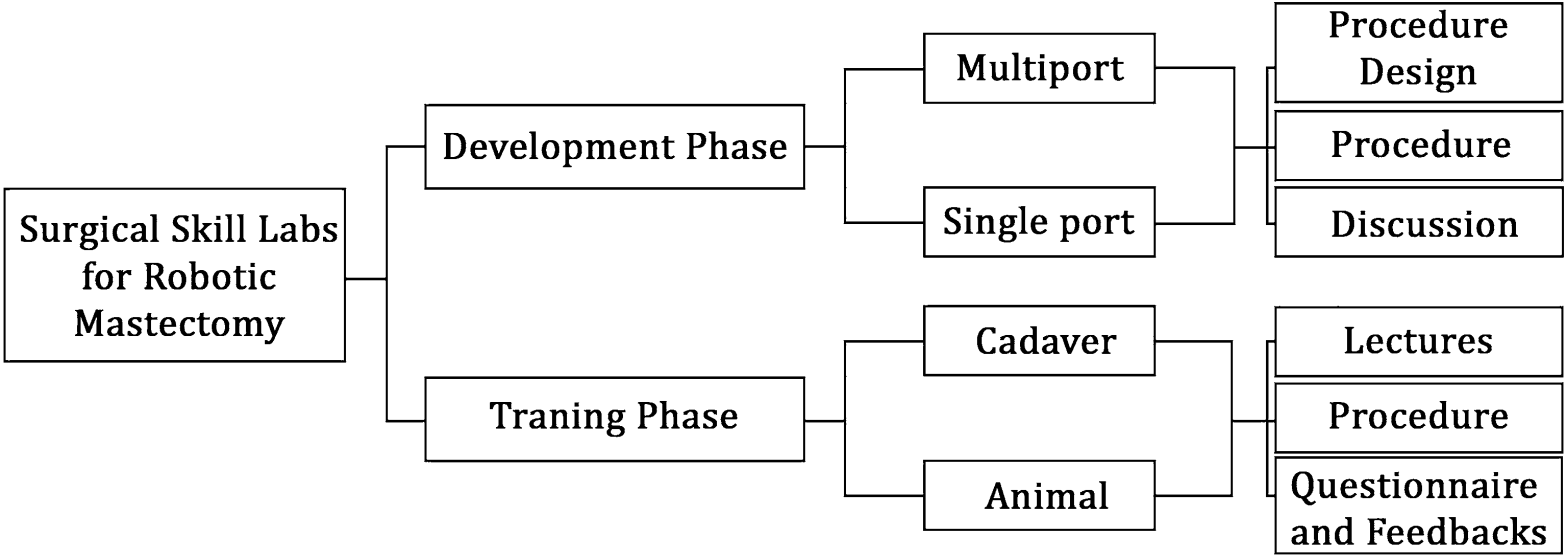
Categories of surgical skill laboratory training programs for robotic mastectomy.

Cadaveric or porcine skill laboratory training was categorized into two phases consisting of two modules each (Fig. 1). The initial development phase consisted of multiport and single port system modules. Its process included the procedure design, procedure, and participant discussion. On the other hand, the training phase consisted of cadaveric and animal modules. Its process included lectures, procedures, questionnaires, and feedback.

The cadaveric skill laboratory training that was performed before the first attempt at multiport or single port RNSM was defined as the multiport or single port system modules of the initial development phase^5,15^. For the initial development phase of the multiport system module, the design and development of a new procedure were established. During the entire procedure, all participants evaluated the feasibility and safety of RNSM. This phase was performed by surgeons who had no experience in RNSM and were under no supervision. The multiport system module in the initial development phase included immediate breast reconstruction using a latissimus dorsi (LD) flap. The reconstruction procedures were motivated by and referred to a study that used a robotic LD flap^14^. Park et al*.* previously described the SP® module^15^, which was designed for a single port surgical system, the da Vinci SP®.

The training modules in the training phases were designed for beginners by an experienced surgeon who developed and actively performed RNSM. Therefore, the training phase included basic lectures about the robotic surgical systems and standard RNSM operating procedures. In this phase, an experienced surgeon functioned as a supervisor, and the whole procedure was lectured by an experienced supervisor. After the education program, the accurate and meticulous feedback given by the supervisor was duly noted and incorporated to help the trainee improve. The training phase included five cadaveric and one porcine training modules for 10 trainees.

**Figure 2:**
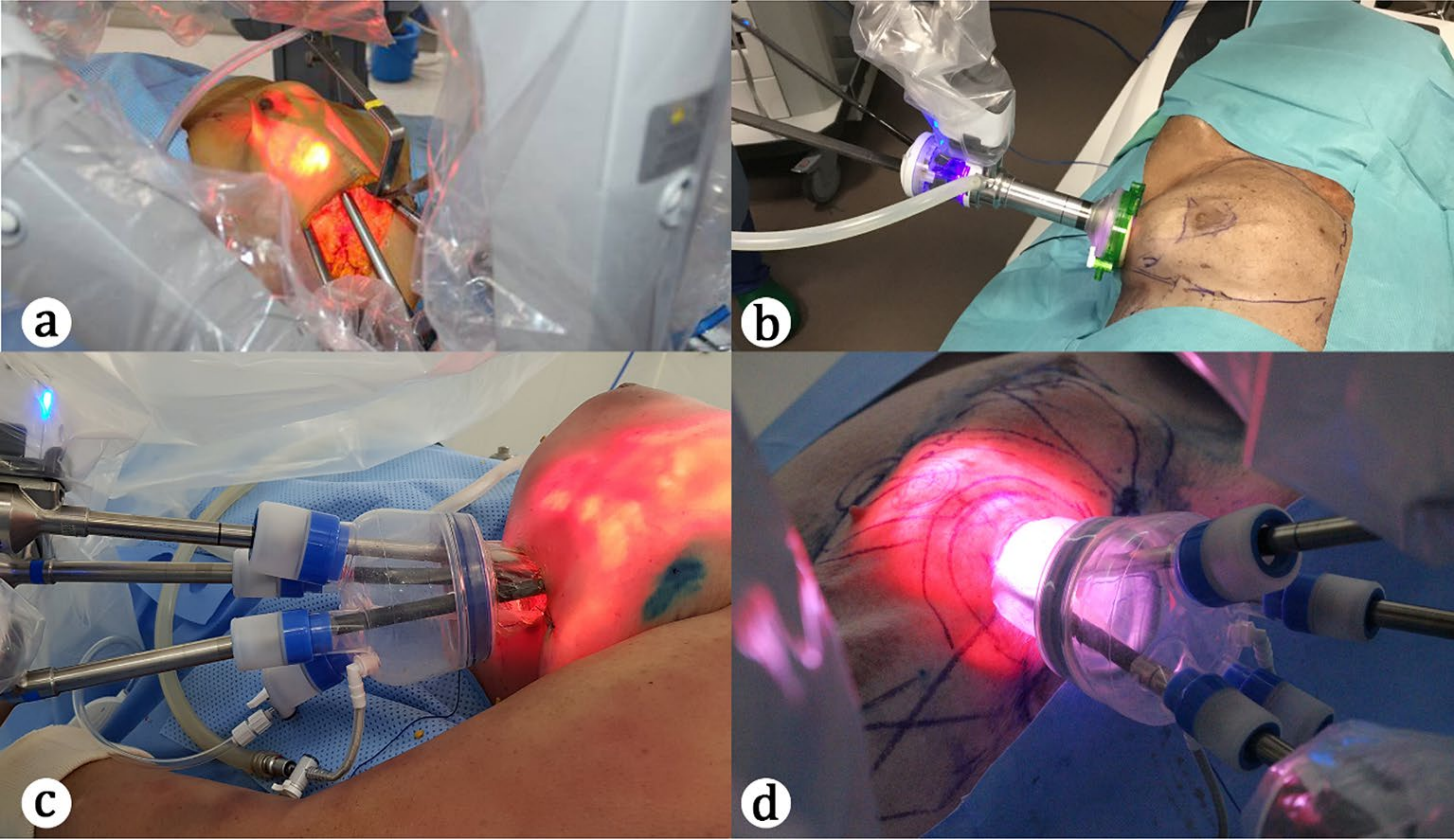
Procedures during various types of surgical skill laboratory training for robotic mastectomy. (**a**) A procedure during the initial development phase of the surgical skill laboratory training for robotic mastectomy using a multiport. (**b**) A procedure during the initial development phase of the surgical skill laboratory training for robotic mastectomy using a single port. (**c**) A procedure during the training phase of the surgical skill laboratory training for robotic mastectomy using a multiport in a cadaveric model. (**d**) A procedure during the training phase of the surgical skill laboratory training for robotic mastectomy using a multiport in a porcine model.

Table 1 shows the general skill laboratory characteristics. For the initial development phases, twelve procedures were performed consecutively on six cadavers. We used both breasts on three cadavers (six in total) for the multiport and single port RNSM development (Fig. 2a). For the multiport RNSM with immediate LD reconstruction development, two breast and two plastic surgeons participated in the multiport system module. There were four procedures performed using the da VinCi Si® system. On the other hand, two procedures were performed using the Xi® systems. Gasless techniques were applied to all RNSM procedures in the multiport system modules in the initial development phase. Unfortunately, there was one injury involving the medial pectoralis muscle and costal cartilage in the first skill laboratory training. Other than this, there were no other intra-operative events or open conversions.

**Table 1:** General characteristics of the skill laboratories of robotic nipple-sparing mastectomy.

Phase	Module	No	Date	Type of model	Robot system	Method	Remarks
Initial development	Multi﻿port	1	Dec. 2013	Cadaver 1	Si	Gasless	LD + implant
		2	Dec. 2013	Cadaver 1	Si	Gasless	LD + implant
		3	Mar. 2014	Cadaver 2	Si	Gasless	LD + implant
		4	Mar. 2014	Cadaver 2	Si	Gasless	LD + implant
		5	Nov. 2014	Cadaver 3	Xi	Gasless	LD + implant
		6	Nov. 2014	Cadaver 3	Xi	Gasless	LD + implant
	Single port	7	Nov. 2018	Cadaver 4	SP	Gas	
		8	Nov. 2018	Cadaver 4	SP	Gas	
		9	Nov. 2018	Cadaver 5	SP	Gas	
		10	Nov. 2018	Cadaver 5	SP	Gas	
		11	Nov. 2018	Cadaver 6	SP	Gas	
		12	Nov. 2018	Cadaver 6	SP	Gas	
Training	Cadaveric	13	Mar. 2019	Cadaver 7	Xi	Gas	
		14	Mar. 2019	Cadaver 7	Xi	Gas	
		15	Sep. 2019	Cadaver 8	Xi	Gas	Implant/ALND
		16	Sep. 2019	Cadaver 8	Xi	Gas	Implant/ALND
		17	Jun. 2020	Cadaver 9	Xi	Gas	Implant/ALND
		18	Jun. 2020	Cadaver 9	Xi	Gas	Implant/ALND
		19	Aug. 2020	Cadaver 10	Xi	Gas	ALND
		20	Aug. 2020	Cadaver 10	Xi	Gas	ALND
		21	Nov. 2020	Cadaver 11	Xi	Gas	ALND
		22	Nov. 2020	Cadaver 11	Xi	Gas	ALND
	Porcine	23	Dec. 2019	Porcine 1	Xi	Gas	
		24	Dec. 2019	Porcine 1	Xi	Gas	

*ALND* axillary lymph node dissection, *LD* latissimus dorsi flaps.

Two breast surgeons participated in the single port system module development (Fig. 2b). At the time of the single port system module, immediate reconstruction was not simulated^15^. A gas-inflated technique using the da Vinci SP® system was used for all procedures in this module.

A total of 12 consecutive procedures with one porcine and five cadaveric models were used for the training phase. Both breasts on the five cadavers (10 in total) were used by 10 trainees. All procedures were performed using a gas-inflated technique using the da Vinci Xi® system with single port devices (Fig. 2c). Immediate direct-to-implant reconstructions were simulated on both breasts of the two cadavers (four in total) by three breast surgeons.

In the porcine model, we divided six breasts and nipples into two procedures: the right and left (Fig. 2d). A total of four breast surgeons participated in the animal skill laboratory training.

The first four cadaveric RNSMs with LD flaps and implants were performed using the da Vinci Si® system. We conducted 14 RNSMs using the da Vinci Xi® system and six using the SP® system. There were 6 gasless and 18 gas-inflated RNSMs that were simulated. A total of 10 RNSMs were followed by reconstruction using the six LD flaps and implants and four direct-to-implants. Robotic axillary lymph node dissection was performed on four cadavers.

We described the general skill laboratory training characteristics, which included the robot systems, subject types (cadaveric or porcine models), gas inflation use, presence of a reconstruction simulation, axillary surgery, and satisfaction of the 10 trainees. A modified questionnaire for basic skill laboratory training from a previous study was used to gauge the trainees’ satisfaction^16^. The questionnaire consisted of three domains: the trainees’ perceived goals in attending the course, teaching/learning environment, and teaching staff performance. The medium of the questionnaire was Google Forms (Mountain View, CA, United States). The satisfaction questionnaire was then assessed by blinding. If a trainee participated several times in the training program, we counted his/her response at the first participation.

### Statistical analyses

This was a pilot study of the development of a new robotic breast surgery. A descriptive statistical analysis was used for general information of the trainees and the training programs, as well as the results of the questionnaires. For each trainee, three scores were calculated as follows: the sum of the ratings of all the variables for each domain was divided by the number of variables in the corresponding domain (session 1—trainees’ perceived goals in attending the course: five variables; session 2—teaching/learning environment: seven variables; session 3—teaching staff performance: six variables; and session 4—comments)^16^. The scores were grouped according to the following values: 1, insufficient/inadequate; 2, reasonable/adequate; 3, sufficient; 4, good; and 5, very good^16^. Continuous variables were described using the mean ± SD and median values with ranges. The SPSS software (SPSS Statistics 25; IBM Corporation, Armonk, NY, United States) was used for statistical analysis.

### Ethics

All cadavers were legally donated to the Yonsei University College of Medicine in South Korea and Medizin im Grünen in Berlin, Germany. The current laboratory procedures at the Surgical Anatomy Education Center, Yonsei University College of Medicine were conducted in accordance with the local regulation, “Act on Dissection and Preservation of Corpses.” Informed consent of all cadavers were obtained from the cadaver donor, who was the next of kin of the cadaver or a legally authorized representative. The animal wet laboratory used one live porcine model for surgical skill training and research. This was approved by the Animal Care and Use Committee of Yonsei University Health System, Korea in accordance with the local regulations, “Laboratory Animal Act (2019-0167).” All experimental protocols of this study were approved by the institutional review board at Severance Hospital (4-2020-1236). All methods were reported in accordance with the Animal Research: Reporting of In Vivo Experiments guidelines. As a pilot study of the development of an educational program, we performed all experimental procedures in accordance with the Declaration of Helsinki, and analyzed the initial experience without randomization or a control group.

## Results

The trainees’ characteristics in the training phase are shown in Table 2. All trainees answered the satisfaction questionnaire. Most of the surgeons who participated in the training phase of the skill laboratory training were younger than 40 years of age (9/10, 90%). Three trainees had more than 50 cases worth of experience in breast surgery at the time of the skill laboratory training. All trainees completed the dry laboratory training using a robot simulator before the skill laboratory training. Seven trainees had previous exposure to robotic breast surgery as observers before the skill laboratory training. Five trainees assisted in RNSMs before the training. Two trainees had experience as operators during robotic surgery before the skill laboratory training.

**Table 2:** General information on the trainees of cadaveric skill lab for robotic nipple-sparing mastectomy.

Variables	Trainees (n = 10)
**Age**	
< 40 years	9
≥ 40 years	1
**Sex**	
Female	5
Male	5
**Clinical experience (number of breast cancer operation)**	
< 50 cases	7
≥ 50 cases	3
**Prior learning of robotic breast surgery (duplicate response)**	
Dry lab (simulator)	10
Attending as an observer	7
Attending as an assistant	5
**Prior experience of robotic breast surgery as an operator**	
No	8
Yes	2

Table 3 shows the scores of the trainees on the three questionnaire domains. Most of trainees gave favorable responses (total median score = 5 [range, 3–5]). The median scores of the delivery of the knowledge required for robotic surgery, and the relationships with the trainees were relatively lower than the median scores of the responses to the other questionnaires (Table 3).

**Table 3:** Modified questionnaires for the trainees of cadaveric skill lab for robotic-assisted nipple-sparing mastectomy (grading scale: 1—insufficient/inadequate; 2—reasonable/adequate; 3—sufficient; 4—good; 5—very good).

Questionnaires	Trainees (n = 10)						
	Scales					Mean ± SD	Median (min–max)
	5	4	3	2	1		
1. Trainees’ perceived goals in attending the course							
(1) Met expectations in knowledge (theory) required for robotic surgery	5	2	3	0	0	4.20 ± 0.919	4.5 (3–5)
(2) Met expectations in skill practice	6	3	1	0	0	4.50 ± 0.707	5 (3–5)
(3) Met knowledge requirements in theory	6	3	1	0	0	4.50 ± 0.707	5 (3–5)
(4) Met the essentials in skills development	8	1	1	0	0	4.70 ± 0.675	5 (3–5)
(5) Developed students at skills performance	6	3	1	0	0	4.50 ± 0.707	5 (3–5)
2. Teaching/learning environment							
(1) Atmosphere of the laboratory	8	1	1	0	0	4.70 ± 0.675	5 (3–5)
(2) Structure of the session	9	0	1	0	0	4.80 ± 0.632	5 (3–5)
(3) Pace of the session	6	2	2	0	0	4.40 ± 0.843	5 (3–5)
(4) Mix explanation/opportunity to practice	6	1	3	0	0	4.30 ± 0.949	5 (3–5)
(5) Trainees’ involvement	7	2	1	0	0	4.60 ± 0.699	5 (3–5)
(6) Development of trainees’ interest in the skills	9	0	1	0	0	4.80 ± 0.632	5 (3–5)
(7) Training adequacy	9	0	1	0	0	4.80 ± 0.632	5 (3–5)
3. Teaching staff performance							
(1) Relationship with trainees	4	5	1	0	0	4.30 ± 0.675	4.5 (3–5)
(2) Enthusiasm for teaching	7	2	1	0	0	4.60 ± 0.699	5 (3–5)
(3) Encouragement of trainee’ participation	6	2	2	0	0	4.40 ± 0.843	5 (3–5)
(4) Clear explanations	7	2	1	0	0	4.60 ± 0.699	5 (3–5)
(5) Clear demonstration of each component of the skill	5	4	1	0	0	4.40 ± 0.699	5 (3–5)
(6) ‘Feedback’	6	2	2	0	0	4.40 ± 0.843	5 (3–5)
4. Comments							
The educational program was necessary to perform robotic surgery. I would like to attend the educational program again It was very helpful to learn the basic knowledge and skills for robotic-assisted nipple-sparing mastectomy							

## Discussion

This study demonstrated that cadaveric and animal skill laboratory training programs for robot-assisted breast surgery fulfilled most trainees’ satisfaction parameters. A few pioneers have recently developed and introduced RNSM; however, most breast surgeons who have no experience with this new system may have concerns about using this robot-assisted surgical system without appropriate education. Therefore, successful application of this innovative surgical system for sophisticated surgical procedures requires appropriate educational programs. Furthermore, the use of RNSM by breast surgeons who do not have the appropriate knowledge of robot-assisted surgical systems may lead to critical errors during surgery. Therefore, the development of effective educational programs for this new innovative surgical procedure is crucial.

In robotic urologic surgery, various educational programs, which included online teaching and a dry laboratory simulator, an operating room, and cadaveric and animal model training, were introduced^17^. There are several previously verified educational methods that may be easy-to-approach and efficient in helping beginners apply robotic surgical systems^2,17^. In this study, most of the trainees reported that they were satisfied with our educational programs in the questionnaire (Table 3). This suggests that our pilot study can be considered the foundation. In addition, we provide initial data on the RNSM training program for breast surgeons who lack experience and knowledge about robotic surgical systems.

Using our pre-clinical models in the initial setting phase, we successfully implemented various techniques that included gas-inflated or gasless RNSM using multiport or single port, robotic axillary lymph node dissection, reconstruction with an LD flap, and/or direct-to-implant in real procedures. Sarfati et al*.* reported similar cadaver-based approaches^18^. Considering the current limitations in RNSM educational programs and training resources for breast surgeons or trainees, our approach suggests that the use of cadaveric skill laboratory training programs can be helpful in obtaining the appropriate knowledge and surgical skills for RNSM.

Here, we developed two RNSM skill laboratory models: porcine and cadaveric. The general features of the two models are summarized in Table 4. In general, at our institution, the porcine models are less expensive and more accessible than the cadaveric models. Since the porcine models are in vivo models, their vascular structures are intact; therefore, the simulation of bleeder ligation and bleeding control in these models is more feasible than that in cadaveric models. However, the anatomical structure similarities are far better in the cadaveric model than in the porcine model. Even though the cadaveric models are incredibly similar to real circumstances, porcine models can also simulate the dissection plane, which is the most important component of the mastectomy procedure. A previous study evaluated the value of porcine versus cadaveric models for procedural training of general surgery residents^19^. It found that anatomic relevance was better in cadaveric than in porcine models, while tissue handling and anatomic plane simulation were better in the porcine model than in the cadaveric model^19^. However, the overall training value did not differ between the two models. Essentially, cadaveric skill laboratory training may provide anatomical similarity and relevance, and can be considered one of the best options for RNSM training. However, due to the limited resources, porcine models may be used as alternatives.

**Table 4:** Comparative features of porcine and cadaveric skill laboratories.

	Porcine	Cadaver
Cost	USD 2500–3000	USD 4500
Availability of specimen	Good	Limited
Simulation of bleeding	Good	None
Volume of breast	Poor	Very good
Anatomic similarity	Poor	Very good
Simulation of dissecting anatomic plane	Good	Very good

There were two questions that received relatively low median scores compared to the other questions. These questions were related to the following topics: the expectations pertaining to the knowledge required for robotic surgery and the relationship between the trainer and the trainees. The expectations pertaining to the knowledge required for robotic surgery can be improved by amending the amount of time spent training and increasing the depth of knowledge provided on robotic mastectomy. Online teaching, evaluation of pre/post-examination, or handbook development may be valuable tools that can facilitate the improvement of the skill training to meet the trainees’ expectations of the knowledge required for RNSM. The relationship between trainers and trainees may be affected by prior relationships between the two parties and their personal dispositions.

This study is important as it provides the basic structure for a standardized RNSM training protocol. In addition, another study suggested that an RNSM training system should be established to accumulate evidence on the feasibility and oncologic outcomes that counteract the warning from the United States Food and Drug Administration safety communication announced in 2019^10^. However, if unskilled surgeons who have not participated in a well-structured training program participate in clinical trials to establish evidence for oncologic outcomes or participate in a real practice, this may result in dangerous consequences for the patients. Therefore, this study is significant as it provides the initial experience of the first RNSM educational program in the initial period of this new procedure. In the near future, we plan to conduct a prospective research study to develop a more effective and standardized educational program using an accurate and objective assessment tool of the RNSM skill improvement.

This study has several limitations. First, only few trainees participated in the training phase. Second, this was a retrospective study. The result of the participants’ answers to the questionnaire may be inaccurate since the participants may have depended on their own memory while responding late to the questionnaire. Third, there was a lack of learning curve analysis and objective training score measurement to assess the effectiveness of the educational program. A prospective study with a larger sample size using other objective measurement analyses of training effectiveness, such as Global Evaluative Assessment of Robotic Skills^20^, is needed to prove the effectiveness of RNSM cadaveric and animal skill laboratory training programs. Nevertheless, to the best of our knowledge, this is the first collective analysis of pre-clinical cadaveric and porcine skill laboratory training programs for RNSM.

In conclusion, we suggest that cadaveric or porcine RNSM skill laboratory training programs is essential in offering safe and efficient training. Therefore, standardized RNSM training protocols should be established.
